# *ApoE* Modifier Alleles for Alzheimer's Disease Discovered by Information Theory Dependency Measures: MIST Software Package

**DOI:** 10.1089/cmb.2022.0185

**Published:** 2023-03-07

**Authors:** Andrew Banman, Nikita A. Sakhanenko, James Kunert-graf, David J. Galas

**Affiliations:** Pacific Northwest Research Institute, Seattle, Washington, USA.

**Keywords:** Alzheimer's disease, ApoE modifier alleles, information theory dependency measures, software package

## Abstract

Information theory-based measures of variable dependency (previously published) have been implemented into a software package, MIST. The design of the software and its potential uses are described, and a demonstration is presented in the discovery of modifier alleles of the *ApoE* gene in affecting Alzheimer's disease (AD) by analyzing the UK Biobank dataset. The modifier genes uncovered overlap strongly with genes found to be associated with AD. Others include many known to influence AD. We discuss a range of uses of the dependency calculations using MIST that can uncover additional genetic effects in similar complex datasets, like higher degrees of interaction and phenotypic pleiotropy.

## 1. INTRODUCTION

Biological systems generate intrinsically complex datasets in which a large set of variables and attributes interact by storing and transmitting information among one another. This information depends on how each variable interacts with and is related to the other variables of the system. This can be summarized by determining how the variables depend on one another. Characterization of the joint probability distribution of the variables is at the heart of describing their mathematical dependency. We have devised measures of dependency of subsets of variables based on information theory (Galas et al, 2021; Galas et al, 2014).

MIST provides a Multivariable Information Theory-based dependence Search Tool, and a new software implementation package, which calculates these measures for complex datasets. It is designed to rapidly compute the joint probability density for many variables, then compute the entropy-based measures designed to detect and characterize the functional dependencies among variables (Galas et al, 2020; Galas et al, 2014). In this context, a function between variables defines a deterministic relationship between them, a dependency; it can be as simple as “*if X then Y*” or much more complicated involving many variables. MIST is designed to quickly find functional dependencies among many variables. It uses model-free Information Theory measures to compute the strength of dependence in any given subset of variables. MIST detects and quantifies functional dependencies for any function, involving any number of variables, limited only by processing capability and statistical power. MIST is therefore a powerful tool for paring down a large set of variables into subsets of dependent variables, which provides biological evidence and may then be studied further.

It is important to emphasize again that since it is based on Information Theory measures, which quantify the dependencies in the data without making any modeling assumptions, MIST does not answer the question of how the variables are dependent on one another. It does not recover the underlying function governing a dependence. On the other hand, MIST establishes the *existence* of a dependence among a set of multiple (two, three, or more) variables. Given the dependence identified by MIST, we can choose to find the function underlying the dependence using a model as simple as logistic regression or more complex, like Bayesian networks. Note also that identifying the subsets of dependent variables is like extracting the structure from the data, which may also be seen as a compression of the data.

In this article, we briefly review the previously published mathematical basis of the dependencies (Galas et al, 2021; Galas et al, 2014), describe the design of the software, and then demonstrate MIST's utility by analyzing genetic and clinical data from the UK Biobank (UKBB) database. Specifically, we begin by searching for pairwise functional relationships between single nucleotide polymorphisms (SNPs) and the Alzheimer's disease (AD) phenotype. We then study new genetic dependencies by finding SNPs that modify a well-known genetic variant (*APOE4*) that affects AD. We also provide a detailed performance analysis of MIST on included test data. The aim of this article is to showcase MIST software that can make the data analysis of large datasets deeper and produce richer results. Although we chose to use genetic data of AD as an example, the same ideas can be applied to other types of data.

## 2. METHODS

### 2.1. Information measures

Given a set of *N* variables and a set of *M* samples representing information on each of the variables, how do we find functional relationships between the variables? MIST tackles the problem by using a divide-and-conquer approach: while considering the set of all variable tuples as the search space, MIST divides that search space among parallel threads, and then conquers it by computing dependency measures for each tuple.

Symmetric Delta (Galas et al, 2020; Galas et al, 2014) is the measure used in a MIST search. The Symmetric Delta is a novel symmetric measure of functional dependence (it is symmetric under exchange of variables) constructed from joint entropies. Joint entropies between variables (using the Shannon entropy (Shannon and Weaver, 1949) defined as the expectation of the logarithm of each element of the joint probability distribution: $H\left(X\right)=-\sum_{i}P\left(x_{i}\right)log\left(P\left(x_{i}\right)\right)$). For variable tuples of size *T* there are *N choose T* tuples in the search space. Thus, the problem is reduced to computing joint probability distributions for a very large number of variable tuples.

Dependence between two variables *X* and *Y* can be directly measured with *mutual information* $I\left(X,Y\right)$, defined as

$$I\left(X,Y\right)=H\left(X\right)+H\left(Y\right)-H\left(X,Y\right),$$

where $H\left(X\right)$ and $H\left(Y\right)$ are single entropies of variables *X* and *Y* and $H\left(X,Y\right)$ is their joint entropy.

A general dependence among three variables, *X*, *Y*, and *Z*, can be measured with *symmetric delta.* $\bar{\Delta }\left(X,Y,Z\right)$. To see clearly the definition of symmetric delta, we need to introduce *interaction information*, which is a multivariable generalization of mutual information (McGill, 1954), defined for three variables as

$$I\left(X,Y,Z\right)=I\left(X,Y\right)-I\left(X,Y|Z\right).$$

Given interaction information, *differential interaction information* Δ is defined as a difference between values of successive interaction information arising from adding a variable:

$$\begin{array}{cc} \Delta_{X} & =I\left(X,Y,Z\right)-I\left(Y,Z\right),\\ \Delta_{Y} & =I\left(X,Y,Z\right)-I\left(X,Z\right),\\ \Delta_{Z} & =I\left(X,Y,Z\right)-I\left(X,Y\right). \end{array}$$

Here $\Delta_{X}$ is called *asymmetric delta* for the target variable *X*. To detect a fully synergistic dependence among a set of variables, we want a single measure, which is symmetric. Consequently, we defined a general measure $\bar{\Delta }$, called *symmetric delta* (or simply *delta*), by multiplying the Δ's with all possible choices of the target variable:

$$\bar{\Delta }\left(X,Y,Z\right)=\Delta_{X}\cdot \Delta_{Y}\cdot \Delta_{Z}.$$

The critical property of this delta measure is that it is zero whenever any of the three variables is independent of the others. It is important to note that the absolute values of the delta measure indicate the degree to which the corresponding variables are collectively interdependent.

### 2.2. Software design

**FIG. 1.**
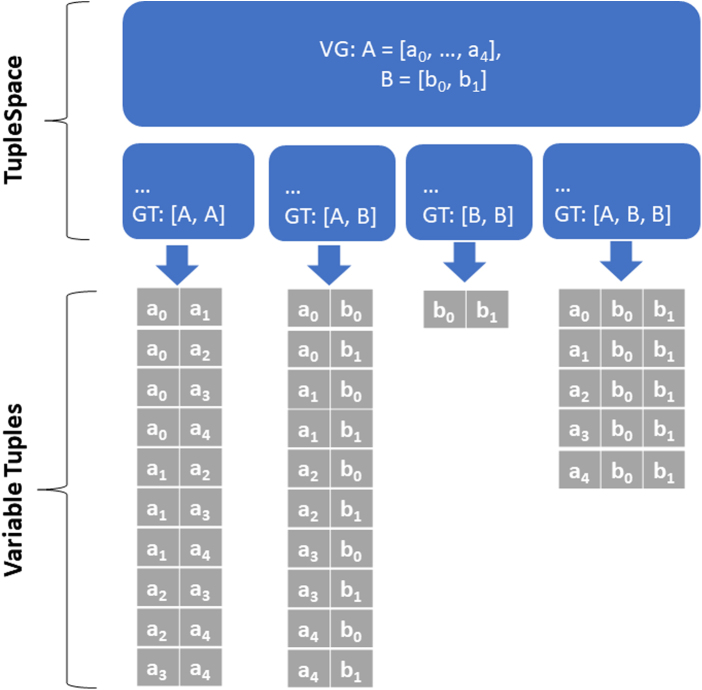
A TupleSpace is composed of VG and GT. The GT act as templates defining the combination of variables drawn from the VG to form Variable Tuples. For example, the Variable at position 0 in the Variable Tuple will be chosen from the group at position 0 in the GT. In this figure, VG A and B are used in four TupleSpaces, each with a different GT. The generated Variable Tuples are displayed underneath each TupleSpace. Since each VG is a disjoint ordered set and each Variable Tuple is an ordered set, Variable Tuples can be determined by simply iterating through the VG. GT, Group Tuples; VG, Variable Groups.

In practice, the bulk of the computational runtime is spent on counting to obtain joint probability distributions, and so we can get a significant speed-up by reducing the number of entropy calculations. First, we avoid doing unnecessary work by carefully defining the search space. For example, it may be that we are only interested in particular combinations of variables, rather than the set of all possible tuples. MIST constructs a *TupleSpace* from user-defined *Variable Groups* and *Group Tuples* that define the sorts of variable tuples to include in the search (Fig. 1). In the AD study below, we reduce one search space size from the order of $10^{16}$ to $10^{11}$ tuples with this constraint.

**FIG. 2.**
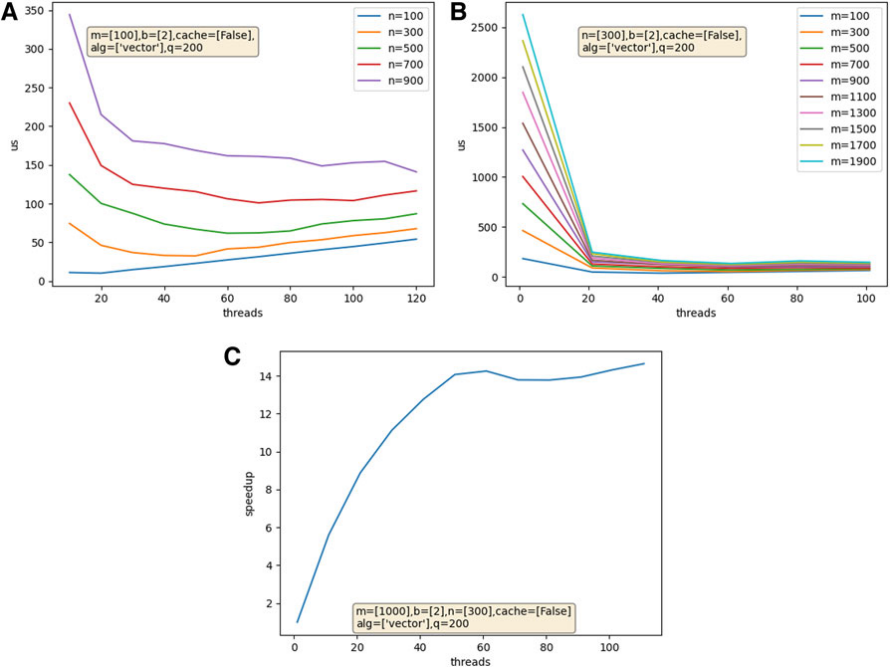
MIST runtime as thread count increases over various N (top, left) and M (top, right) values. The speedup over single core performance (bottom) shows the diminishing returns of thread counts >60. Timings were taken on a server equipped with 4 Intel Xeon Gold 6148 CPUS (total 80 core, 160 threads) and 250GiB RAM. Each measurement was repeated *q* = 200 times and the mean is used.

Next, MIST parallelizes the search by dividing the TupleSpace among computation threads. It uses a traversal algorithm to divide the search space into equally sized, contiguous working sets. By treating the search space as an ordered list of tuples, each thread can skip to the starting position in its working set. Thus, MIST achieves scalable parallelization without interprocess communication. As a result, threads can run on separate systems without coordination software like MPI. Generally, increased thread counts reduce runtime, but with diminishing returns as the nonparallel portion of the runtime, plus any threading overhead, take on a larger portion of the runtime (Fig. 2).

The number of entropy calculations is also reduced by reusing subcalculations in Symmetric Delta. MIST does this by keeping an in-memory cache of joint entropies to be reused in later computations. For example, the Symmetric delta of variables A, B, and C involve the joint entropies *H(A,B)*, *H(A,C)*, and *H(B,C).* If any of these entropies were previously computed, they are retrieved from the cache.

**FIG. 3.**
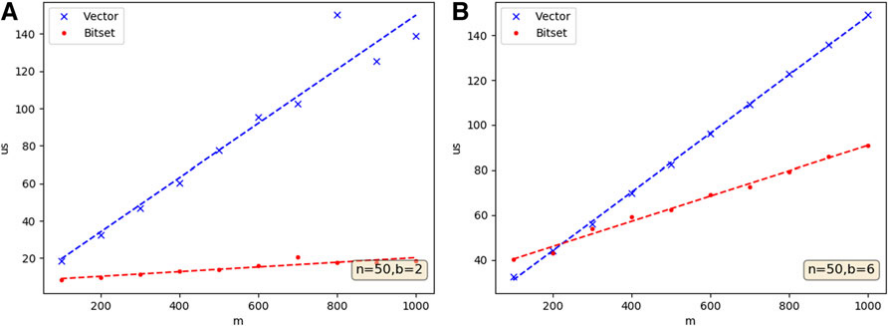
**(A, B)** Runtime performance of randomly generated data consisting of N variables of length M. In most cases the bitset algorithm outperforms the vector algorithm. For data with a large number of value bins **(B)**, the Vector algorithm is faster for some smaller variable sizes. Timings were taken on a workstation equipped with Intel Core i7-6850K and 62GiB RAM.

Finally, we can reduce the computation time of each entropy calculation by exploiting the computer architecture. In MIST's probability distribution “bitset” algorithm, each variable is recast as *B* bitsets of length *M*, where *M* is the number of samples and *B* is the number of bins (number of unique discrete values) of a variable. Each bitset *b_i_* corresponds to one value *x_i_*, where each bit is 1 when that sample has value *x_i_* and 0 otherwise. Then, each element in the probability distribution is the count of 1's in the bitswise AND combination of bitsets: $P\left(x_{i},y_{j}\right)=count\left(b_{i}ANDb_{j}\right)$, where *b_i_* and *b_j_* are bitsets of $X=x_{i}$ and $Y=y_{j}$. This computation is highly compacted, for example, for modern x86_64 architecture CPUs, 64 bits can be processed in one CPU instruction, whereas each integer comparison is a separate instruction. However, this approach is sensitive to the number of value bins and the tuple size, since both dimensions magnify the number of counting operations (Fig. 3).

Keeping the number of tuples constant, the runtime is linear in *M* with a coefficient *B^d^*, where *d* is the number of variables in a tuple, assuming all variables have the same number of bins. Thus, the bitset algorithm is excellent at speeding up searches over variables with few bins and many samples.

## 3. RESULTS

We illustrated MIST on generated test data and real AD diagnosis data from the UKBB.

### 3.1. Test data

The MIST GitHub repository includes a script to create sample data and run three small experiments. The test data contains 90 variables and is specially constructed to hold functional dependencies. These functional dependencies were detected by MIST: a strong *f* (X) = 2*X dependency between one third of the variables, a weaker dependency for the same function among another third of variables, and a three-way f(X,Y) = XOR(X,Y) among the remaining third. The expected results are packaged with the test data. See (Galas et al, 2021; Galas et al, 2014; Sakhanenko and Galas, 2015) for more examples of the MIST procedure.

Scripts to produce the performance measurements in Figures 1–3 are also included in the repository.

### 3.2. APOE modifiers in AD

We now present an example of how the measures of variable dependence can be used to establish relationships among variables in a dataset. The power of the measures is rather general and can be used in several ways, some of which we describe in a little more abstract, general way. In this study, we demonstrate a specific example by determining a set of modifier alleles of a known allele that predisposes to AD using the data of the UKBB. Note that the AD example is just that, an example of MIST application, and although it produced some biologically very interesting results, it was not our intention to be exhaustive in the analysis of AD so as not to detract from showcasing the MIST software. We piggyback on thorough and vast mathematical theory behind MIST that includes comparisons to other measures of variable relationships (Reshef et al, 2011) or the effects of data features, such as the case–control structure or the minor allele frequency, on the information measures (Ignac et al, 2014). Here is a brief description of the strategy: Suppose we know a pairwise connection of variables like the *apoE4* SNP and the AD phenotype.

We can then use the three-way symmetric delta measure (we will call it D3 here) to find any variables that are codependent with these two. This is done by calculating D3 for the three variables (phenotype AD, SNP *apoE4*, and any other SNP). These triplets are then ordered by their D3 scores and the SNPs that are included in the high scoring triplets with the other two (*apoE4* SNP and AD) are thus identified as codependent with them. Since the new SNPs modify the dependence between the original SNP and the phenotype, these are modifier alleles.

From the UKBB, we retrieved 2778 individuals that were diagnosed with AD by selecting all individuals with a recorded first AD diagnosis occurrence (data field 131036: date ICD-10 code G30 reported). The AD phenotype variable is coded 1 for these individuals. For the control group, we selected random individuals with the same age and sex distribution as the AD set. The control group is coded 0 in the AD phenotype variable. Altogether there are 5556 individuals. Although in this example we chose to keep the cases and controls balanced, we could choose a different strategy depending on the problem, for example, we could increase the number of matched controls in an attempt to increase the association power. We have thoroughly reviewed the effects of the case–control structure of the data on the ability to detect a dependency in our previous publications (Ignac et al, 2014). We want to emphasize again that the choice of the case–control structure is problem dependent and is up to the research; MIST provides the vehicle to solve the problem.

There are 784,256 SNPs in the UKBB for the AD and control sets. Each SNP has *M* values and is coded 0, 1, or 2 for major, minor, and heterozygous alleles. The code-1 is given for subjects that do not have that SNP information in UKBB. Missing values create noise in the data, so we pruned away SNPs with more than 1000 missing values, leaving 767,107 SNPs. Then the data are composed of *N* = 767,107 + 1 variables, each with *M* = 5556 measurements.

We start our calculation by computing the pairwise Mutual Information between AD and every SNP. This yields genetic association results somewhat similar to GWAS. For the SNPs with high Mutual Information, we calculate a permutation-based *p*-value. Specifically, for each SNP we calculated Mutual Information with AD after a permutation such that the linkage between SNPs would be preserved, but the dependence with AD would be randomized. We repeated this process 10,000 times and estimated a *p*-value. More details on permutation testing can be found in our earlier publications (Ignac et al, 2014; Uechi et al, 2020). Although, we capped this process at 10,000 permutations to save resources, it can be increased to get a better estimate of a *p*-value (assuming we have enough data). Note that this *p*-value is not adjusted for multiple hypothesis testing, which is suitable for the exploratory analysis shown in our example. While we know that the APOE alleles should be high scorers, in this study, we carry out the full calculation to demonstrate the full use of MIST.

Table 1 shows SNPs from each chromosome with the strongest association with AD using the Mutual information. Note that 16 of these SNPs have a *p*-value of <0.0001. Table 2 shows the location and nearest genes for the strongest SNPs, and provides literature indicating the connections to AD.

**Table 1.** Single Nucleotide Polymorphisms with the Highest Mutual Information with Alzheimer's Disease for Each Chromosome

chr	Snp	mi	*p*	maj-ad	min-ad	het-ad	maj-ctrl	min-ctrl	het-ctrl	*n*
19	rs429358	0.109292	0.0001	863	1121	350	1693	590	46	4663
9	rs150116611	0.00379278	0.0001	2776	0	0	2755	21	0	5552
1	rs639233	0.00363657	0.0001	2088	593	91	2013	711	45	5541
11	rs12418255	0.00348438	0.0001	2607	169	0	2676	96	4	5552
5	rs115512732	0.00327271	0.0001	2682	91	0	2613	159	4	5549
20	rs74372499	0.00314683	0.0001	2280	448	48	2171	574	27	5548
12	rs61739220	0.00298809	0.0001	2670	106	0	2727	48	0	5551
10	rs77145664	0.00291229	0.0001	2523	252	0	2500	260	16	5551
4	rs10006295	0.00287525	0.0001	2462	284	19	2451	321	1	5538
16	rs8052709	0.00282347	0.0001	128	852	1795	123	1015	1636	5549
7	rs4960602	0.00280291	0.0001	2195	535	46	2298	462	17	5553
21	rs9978204	0.00273441	0.0001	888	1436	445	985	1267	517	5538
18	rs1437070	0.0027177	0.0001	1108	1045	328	956	1173	332	4942
22	rs2142833	0.00267846	0.0001	716	1293	761	624	1459	683	5536
3	rs1433349	0.00261743	0.0001	704	1446	603	767	1284	703	5507
13	rs73239750	0.00238418	0.0001	2652	65	2	2620	117	0	5456
17	rs9909573	0.00273371	0.000882454	1699	679	107	1798	599	64	4946
14	rs61094873	0.00277542	0.002471764	2598	151	26	2653	114	5	5547
6	rs11752643	0.00662105	0.027549171	582	24	5	659	44	2	1316
8	rs11776689	0.00475156	0.047212436	719	108	1	717	87	10	1642
2	rs72799081	0.00521001	0.174050867	425	75	7	448	60	1	1016
15	rs35414723	0.00508569	0.178271327	400	70	12	409	104	15	1010

Ordered by *p*-value. SNPs with *p*-value 0.0001 are ordered by mutual information value.

**Table 2.** Single Nucleotide Polymorphisms with *p*-Value 0.0001, Their Nearest Genes and Their Relevance to Alzheimer's Disease

chr	snp	mi	*p*	Location	Relevance and references
19	rs429358	0.1093	0.0001	exon of APOE	One of the two SNPs that determine 3 common variants of ApoE gene, and in particular the ApoE e4 variant, which is a risk variant for AD (Farrer et al, 1997; Roses, 1994)
9	rs150116611	0.0038	0.0001	exon of HRCT1: Missense Variant	A possible role in modulating neurotransmitter activity in the liver, kidney, and brain, and their clearance from the blood (Snieder et al, 2019); one of the genes indirectly connected with AD and Type 2 diabetes (Hu et al, 2020)
1	rs639233	0.0036	0.0001	exon of PTP4A2: Non-Coding Transcript Variant	Involved in molecular pathways involved in neurodegenerative disorders (von Schantz et al, 2008)
11	rs12418255	0.0035	0.0001	Intron of LINC02713	No known significance with respect to AD
5	rs115512732	0.0033	0.0001	Intron of LOC105377693, ATP10B (134 KB), GABRB2 (224 KB)	A key player in neuropsychiatric disorders (Barki and Xue, 2022)
20	rs74372499	0.0031	0.0001	Intron of SULF2	Implicated in Alzheimer's disease pathogenesis (Roberts et al, 2017)
12	rs61739220	0.0030	0.0001	Exon of BCL2L14: Missense Variant	Part of the complex associated with Alzheimer's disease (Sharma et al, 2017)
10	rs77145664	0.0029	0.0001	RTKN2 (4 KB), ZNF365 (101 KB)	Involved in a cytoprotective signaling pathway downregulated in AD brains (Gongol et al, 2017)
4	rs10006295	0.0029	0.0001	AADAT (699 KB)	Involved in thyroid hormone regulation, which is associated with a risk factor of AD (Li et al, 2021; Teumer et al, 2018)
16	rs8052709	0.0028	0.0001	Exon of CIITA: Non-Coding Transcript Variant	Attenuates inflammation and neurodegeneration (Williams et al, 2018)
7	rs4960602	0.0028	0.0001	Intron of DPP6	Involved in early-onset AD (Cacace et al, 2019)
21	rs9978204	0.0027	0.0001	MRPL39 (265 KB)	Involved in mitochondrial dysfunction, which is a consistent feature of AD in the brain and blood (Lunnon et al, 2017; Nazarian et al, 2019)
18	rs1437070	0.0027	0.0001	Intron of WDR7	A hub gene associated with mitochondrial dysfunction and synaptic loss (Miller et al, 2008)
22	rs2142833	0.0027	0.0001	Intron of APOBEC3B-AS1, APOBEC3B (14 KB)	Involved in RNA editing implicated in AD (Christofi and Zaravinos, 2019)
3	rs1433349	0.0026	0.0001	WNT7A (20 KB)	Involved in Wnt signaling deregulation in AD (Palomer et al, 2019)
13	rs73239750	0.00238418	0.0001	NDFIP2 (162 KB)	One of the diabetes-related genes with altered expression in AD brains (Hokama et al, 2014)

AD, Alzheimer's disease; SNPs, single nucleotide polymorphisms.

We use a TupleSpace object to limit the search from all possible variables to only tuples containing one SNP and the AD phenotype variable. This reduces the number of tuples to compute from the default 2.9 × 10^11^ to 7.6 × 10^5^. The API calls to create this tuple space are:

ts = mist.TupleSpace()

g1 = ts.addVariableGroup(“snps”, snps)

g2 = ts.addVariableGroup(“ad”, ad)

ts.addVariableGroupTuple([g1,g2])

**FIG. 4.**
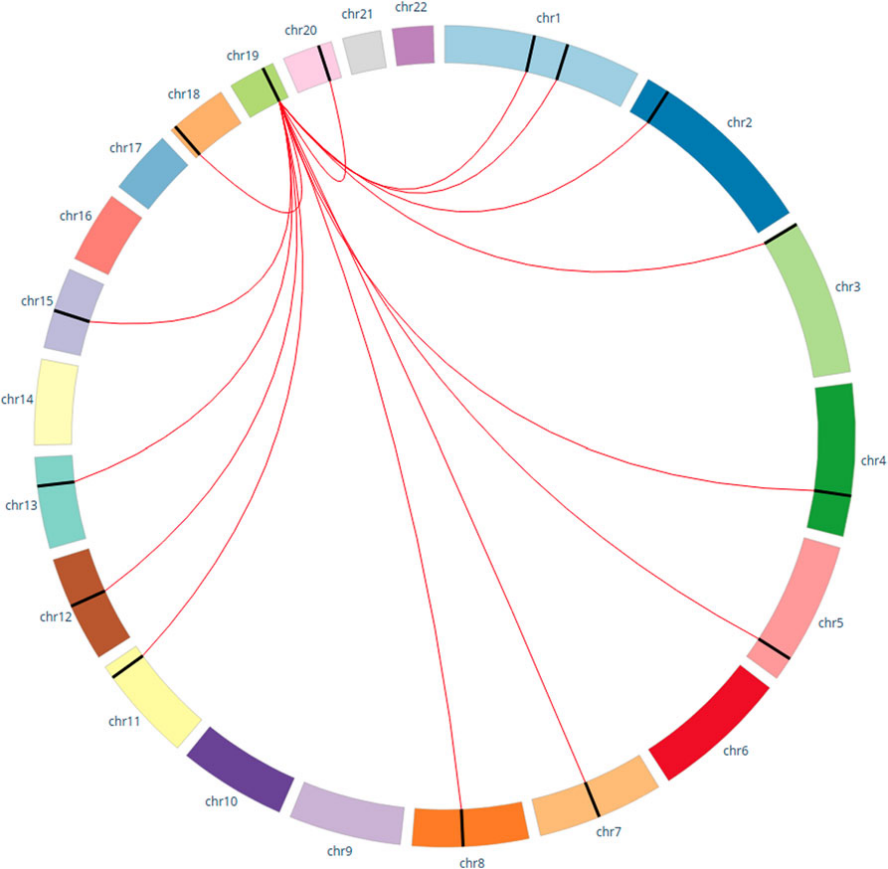
ApoE modifiers (ApoE is shown on Chr 19). Black lines mark the 10 top-scoring primary genes (top mutual information scores between SNPs and AD phenotype). The red lines indicate the top-scoring modifiers (Tables 3 and 4). AD, Alzheimer's disease; SNPs, single nucleotide polymorphisms.

We then calculate a 3-way measure Delta (AD, *APOE*, SNP), where *APOE* corresponds to the SNP rs429358, the risk variant for AD. The 3-way measure is calculated for each SNP. We selected 200 SNPs with the largest Delta values and calculated unadjusted permutation-based *p*-values. Note that we permuted variables AD and *APOE* to test the hypothesis that we cannot achieve large Delta values with random pairwise dependencies among AD, *APOE*, and SNP. For more information about different strategies of permutation-based *p*-value estimation see Ignac et al, 2014. Table 3 shows SNPs with the strongest 3-way dependence with AD and APOE. Note that four of these modifier SNPs have a *p*-value of <0.0001. Note that these SNPs modify the dependency between the *APOE* SNP and the AD phenotype, so these are modifier alleles. Table 4 and Figure 4 show the location and nearest genes for the strongest SNPs, and notes the literature indicating the connections to AD.

**Table 3.** Modifier Single Nucleotide Polymorphisms with the Highest Delta3 Computed with Alzheimer's Disease and APOE and Lowest *p*-Value

chr	snp	d3	*p*	mi-snp-apoe	mi-snp-ad	mi-apoe-ad
5	rs76540439	1.90E-05	0.0001	0.02204359	0.00022746	0.10929242
20	rs74934901	1.13E-05	0.0001	0.01461414	0.00145989	0.10929242
1	rs1395562	1.06E-05	0.0001	0.01607514	0.00010976	0.10929242
12	rs79904286	1.01E-05	0.0001	0.01859569	0.00017447	0.10929242
3	rs1145043	1.09E-05	0.00135647	0.009083	0.00016035	0.10929242
8	rs117560288	9.50E-06	0.00147173	0.01295714	0.00044478	0.10929242
15	rs150484536	1.42E-05	0.00154919	0.01831205	4.97E-05	0.10929242
4	rs17050640	1.13E-05	0.0030107	0.01696611	0.00045297	0.10929242
1	rs2800904	1.00E-05	0.00328276	0.01219154	0.00019551	0.10929242
18	rs147676957	9.82E-06	0.00348387	0.00601588	0.00114659	0.10929242
7	rs73161207	2.12E-05	0.00398856	0.02322554	0.00112284	0.10929242
11	rs7952138	1.06E-05	0.0060364	0.0153944	0.00053817	0.10929242
13	rs9601030	2.30E-05	0.00771234	0.0171492	0.00044575	0.10929242
2	rs77543953	1.67E-05	0.00856936	0.01720461	0.00016264	0.10929242

Ordered by *p*-value. SNPs with *p*-value of 0.0001 are ordered by D3 values.

**Table 4.** Delta3 (Modifier) Single Nucleotide Polymorphisms with *p*-Value 0.0001, Their Nearest Genes and Their Relevance to Alzheimer's Disease

chr	snp	d3	*p*	Location	Relevance and references
5	rs76540439	1.90E-05	0.0001	NMUR2 (80 KB)	One of differentially expressed genes in AD (George et al, 2022)
20	rs74934901	1.13E-05	0.0001	Intron of SULF2	Implicated in regulation of neuronal cell signaling (Roberts et al, 2017)
1	rs1395562	1.06E-05	0.0001	Intron of KCNN3	Evidence for involvement in neurodegeneration (Trombetta-Lima et al, 2020; Dolga et al, 2012)
12	rs79904286	1.01E-05	0.0001	Intron of ZFC3H1	Evidence of being a regulator of AD progression Aubry et al, 2015)

Similar to the pairwise case, we use a TupleSpace to limit the search to 3-tuples containing one SNP variable, the *APOE* variable, and the AD variable. The API calls to create this tuple space are:

ts = mist.TupleSpace()

g1 = ts.addVariableGroup(“snps”, snps)

g2 = ts.addVariableGroup(“ad”, ad)

g3 = ts.addVariableGroup(“apoe”, apoe)

ts.addVariableGroupTuple([g1,g2,g3])

It is important to point out that there are many methods that aim to detect genetic interactions. For example, one could use standard logistic regression with an interaction term to model the modifier effect of an SNP on *APOE*. In this article, the dependencies were detected by MIST without any modeling assumptions, which allows us to detect not only all the linear effects, but also nonlinear ones that are not captured well by the standard methods. Moreover, MIST allows us to go beyond pairwise effects and detect dependencies among multiple variables (see more in the discussion section). There is a large body of research comparing the information theory-based measures with the standard methods, such as those based on correlation (Ignac et al, 2014; Reshef et al, 2011), and is outside the scope of our article.

## 4. DISCUSSION

The MIST software package provides tools to calculate measures of multivariable dependence based on the symmetric delta information theory measures (Galas et al, 2021; Galas et al, 2014). These can be used in many different ways to discover codependency links between variables in complex datasets. The power of the measures is very general. These measures can also be used to formulate some of the key relationships of quantitative genetics (Galas et al, 2021). We have demonstrated the power of MIST in a specific example by determining a set of modifier alleles of a known allele that predisposes to AD in the large dataset of the UKBB (Sudlow et al, 2015). Starting with a pairwise connection of variables like the causative *apoE4* SNP and the AD phenotype. We then used the three-way measure (D3) to find any genetic SNP variables that are codependent with these two simply by calculating D3 for the three variables (phenotype AD, SNP *apoE4*, and any other SNP). These triplets were ranked by their D3 scores and the new SNPs included in the high scoring triplets with *apoE4* SNP and AD are identified as codependent with them. These are classed as modifier genes/alleles as shown in Tables 3 and 4 and are known in the literature to represent additional AD-affecting genes as shown in the references.

**FIG. 5.**
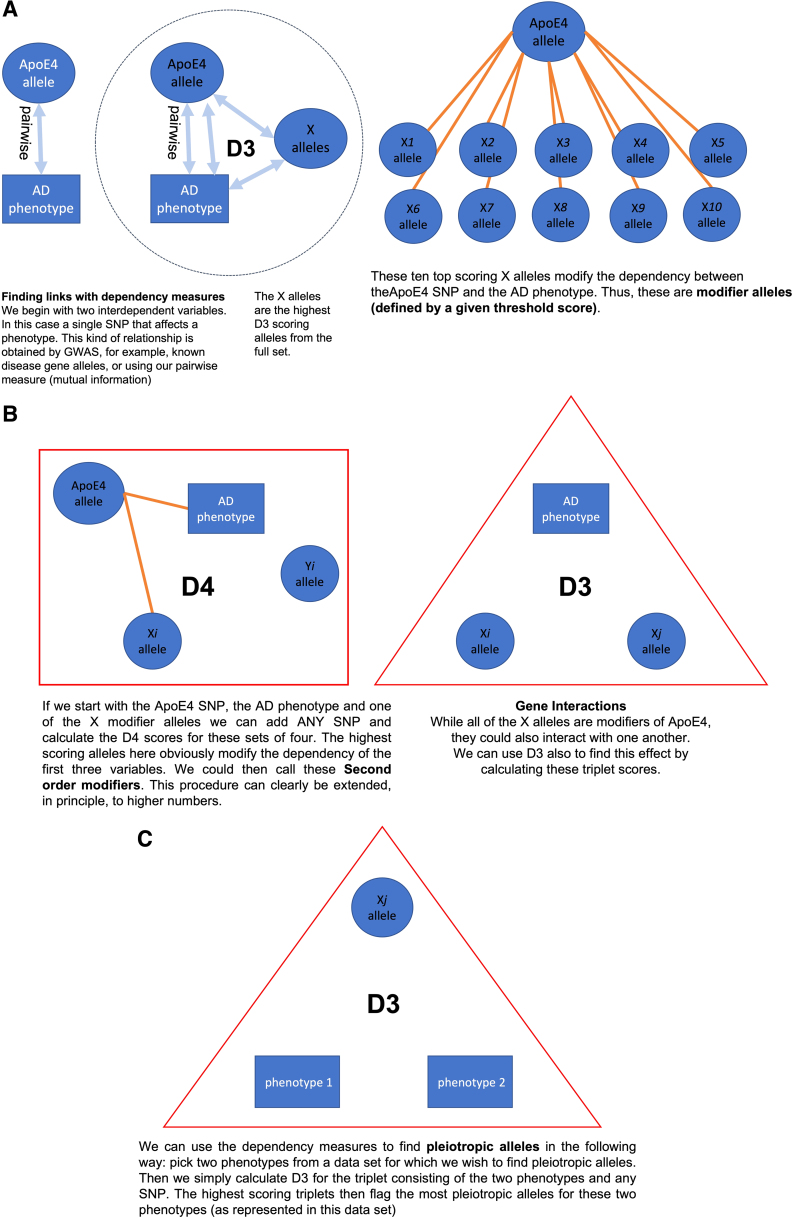
Schematic diagrams of the use of the dependency measures in several analyses of genetic data. **(A)** The search for modifier alleles. **(B)** The search for gene interactions and for second-order modifier alleles. **(C)** The search for pleiotropic alleles.

Some of these identified genes represent interesting factors whose mechanistic, biological roles are yet to be determined. There are a number of other analyses besides the modifier allele discovery that can be performed with MIST. In Figure 5, we diagrammatically summarize the range of simple, direct analyses. To discover modifier alleles, we need to start with a genetic variant for which we wish to find modifiers. In our example we chose the known *APOE* allele, however, in principle any allele with a phenotypic effect can be used if we have a dataset that is appropriate. In Figure 5, Panel A, we depict the calculations schematically using the *APOE4* example (find Allele *APOE4*, find high scoring triplets with *APOE4*, AD phenotype, and a modifier-called {X_i_}). Going further we could, as shown in Panel B, search for two additional effectors by using modifiers and the measure D4 to see if there are additional codependencies among SNPs. In this case, the four-variable set would include the AD phenotype, the *apoE4* SNP, a SNP from the D3 set, {X_i_}, and any other SNP, Y.

These high-scoring SNPs, {Y_i_}, we can term second-order modifiers. We might also look for interactions between any two SNPs from any of the above analyses, which is done by applying D3 calculations. Another biologically interesting analysis would be to start with two different phenotypes and search for a third variable, a SNP that induces a good D3 score (Fig. 5, Panel C). Such SNPs are pleiotropic for the two phenotypes. A little thought about possible multivariable dependencies can yield a number of biologically interesting genetic results. These analyses are certainly not limited to genetics. For example, datasets with phenotypes and biochemical or medical data can lead to interesting sets of biomarkers.

MIST is a general tool for analysis. The model-free search up to four dimensions allows for many kinds of analyses. MIST is designed to take on large problems; its parallel algorithm makes it cluster ready, and targeted search spaces help to filter noise from the results.

### 4.1. Data availability

We utilized generated test data, and real AD diagnosis data from the UKBB. The MIST GitHub repository includes a script to create sample data and run three small experiments. The UKBB AD data comprised individuals with a recorded first AD diagnosis occurrence (data field 131036: date ICD-10 code G30 reported), and a control group of randomly selected individuals with the same age and sex distribution as the AD set. The UKBB data are subject to the legal limitations of a Material Transfer Agreement and may not be shared publicly. To contact UKBB (https://www.ukbiobank.ac.uk/) directly regarding data access, visit https://www.ukbiobank.ac.uk/learn-more-about-uk-biobank/contact-us and select “Enquire about access” to contact the Access Management Team.

The MIST source code is available under an MIT license on GitHub by the name mist. The main library is C++ and links to Boost libraries, such as the Boost::Python library to extend to the python language. MIST also includes a CLI program that can run simple searches and exemplifies the library API. Alternatively, MIST can be run from python using the libmist package available on PyPi or through a Docker image that may be built with the Dockerfile included with the source.

### 4.2. Future directions

The software can be improved in various ways. First, we may add more dependency measures to study relationships in other ways. The module software design allows for easy implementation of other measures to compute for each tuple. We are also interested in allowing the user to define their own measures, rather than having to implement them in the library.

The quality of MIST search results may be improved by including more statistical information along with raw signal strength. For example, provide the option to compute statistical measures, such as *p*-value, during the search rather than after the fact. This would help pare down results based on statistical significance rather than signal strength, which is not always the appropriate filter.

MIST can be further optimized by taking advantage of different computational platforms, such as GPU computing. We expect that the calculation of probability distributions could be translated into CUDA library operations. Optimizations in this module could yield a large benefit in runtime.
